# Fruit characteristics, phytochemical and antioxidant properties of blueberry ash (*Elaeocarpus reticulatus*)

**DOI:** 10.1016/j.heliyon.2018.e00834

**Published:** 2018-10-05

**Authors:** Quan V. Vuong, Hong Ngoc Thuy Pham, Hang T. Vu, Trung Thanh Dang, Thanh Van Ngo, Anita C. Chalmers

**Affiliations:** a School of Environmental and Life Sciences, University of Newcastle, NSW, Australia; b Department of Food Technology, Faculty of Food Technology, Nha Trang University, Viet Nam; c Faculty of Food Science and Technology, Vietnam National University of Agriculture, Viet Nam; d Vietnam National University of Forestry, Hanoi, Viet Nam

**Keywords:** Food science

## Abstract

Blueberry ash (*Elaeocarpus reticulatus* Sm.) fruit has potential for human nutrition, but there is limited information on this fruit. This preliminary study aimed to characterise blueberry ash fruit and examine the influence of extraction solvents on its phytochemical and antioxidant properties. Blueberry ash fruit is dark blue in colour and is a stone fruit of small size (7 mm) and light weight (0.2 g). However, it has a high portion of flesh (60% of fruit weight), which is edible and can be a potential source of phytochemicals. Water, ethanol, acetonitrile, acetone, and their combination were tested for extraction of phytochemicals from flesh of this fruit. Water or absolute organic solvent was ineffective for extraction of phenolic compounds from this fruit, but mixtures of water and organic solvents were more effective, and 50% acetone was the most suitable extraction solvent. Extraction with 50% acetone, this fruit was found to contain high levels of total phenolic content, flavonoids, proanthocyanidins, and anthocyanins (104 mg GAE/g, 155 mg RUE/g, 78 mg CE/g, and 119 mg CGE/g, respectively). In addition, this fruit was found to possess potent antioxidant properties. Therefore, this fruit should be further studied for identification of its phenolic compounds and further tested for their biological properties.

## 1 Introduction

**Fig. 1:**
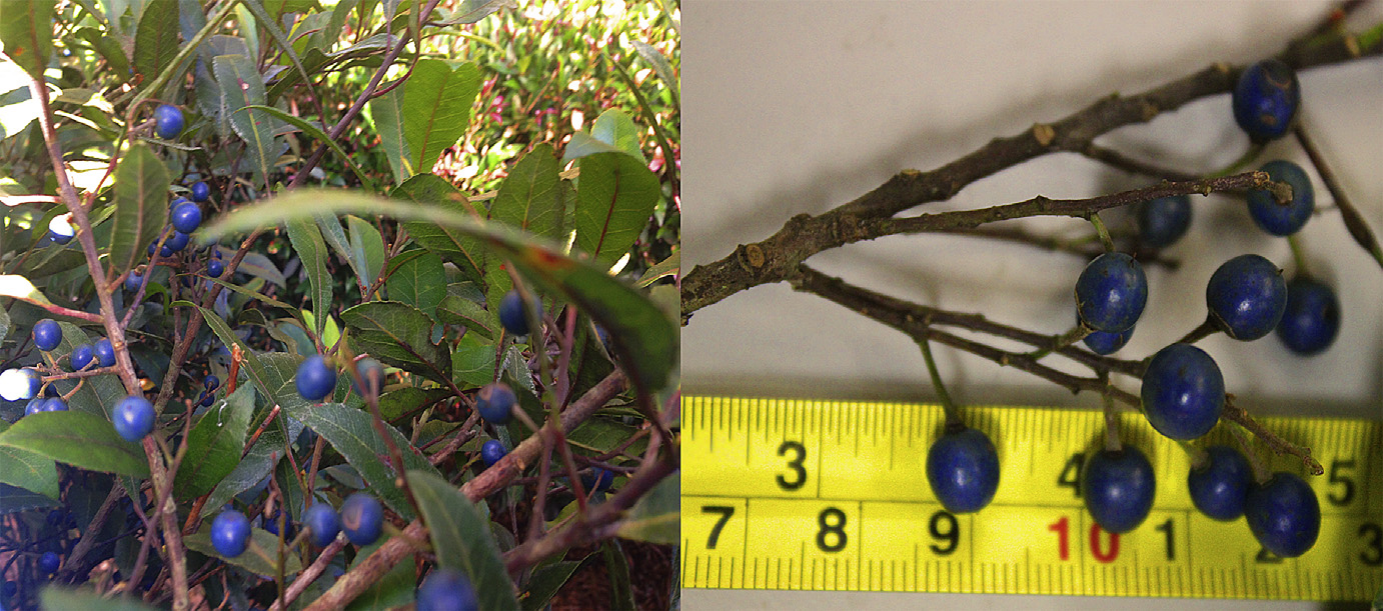
Blueberry ash (*Elaeocarpus reticulatus*) fruit.

Blueberry ash (*Elaeocarpus reticulatus* Sm.) belongs to the family Elaeocarpaceae and is an indigenous shrub or small tree growing in rainforest and coastal scrub along the east coast of Australia, from southern Queensland to Flinders Island in Bass Strait [1]. Blueberry ash fruit are a blue, ovoid to globose drupe (Fig. 1) with a single large endocarp (i.e. stone containing seed). Fruit ripen over many months from December to June [2]. Fruit are eaten by a number of rainforest bird species [3], are edible [4] and have potential for human nutrition [5]. However, there is limited information on the phytochemical and antioxidant properties of blueberry ash fruit.

Extraction solvents have been reported to significantly affect extraction yields of phytochemicals and antioxidant properties in plant materials, such as macadamia husks [6], as well as papaya [7], *Limnophila aromatica* [8], and *Helicteres hirsuta* Lour. leaves [9]. The reason for this is that extraction solvents have different polarities, which selectively extract hydrophilic or hydrophobic phytochemicals from the material [7, 8, 9]. Therefore, it is important to examine the extractability of phytochemicals from blueberry ash using various extraction solvents to identify the most suitable solvent for characterising its phytochemical and antioxidant properties.

As limited information can be found on the characteristics and quality of blueberry ash fruit, this study, for the first time, characterised blueberry ash fruit and examined its phytochemical and antioxidant properties for potential use in nutraceuticals.

## 2 Materials and methods

### 2.1 Materials

Blueberry ash (*Elaeocarpus reticulatus* Sm.) fruit held on stems were collected in August 2015 from the Central Coast region of New South Wales (NSW), Australia (latitude of 33.4° S, longitude of 151.4° E). The plants were authenticated by one of the authors (A.C.C.) and a voucher specimen deposited in the University's Don McNair Herbarium (accession no. 10531).

Once harvested, a portion of fresh fruit was used for characterization. The rest of the fruit were then immediately frozen in liquid nitrogen and freeze dried (FD3 free dyer, Thomas Australia Pty. Ltd., Seven Hills, NSW, Australia) to minimise oxidation or degradation of bioactive compounds. The dried fruit was then ground using a commercial blender (John Morris Scientific, Chatswood, NSW, Australia). The dried fruit flesh was ground and separated from the stone (hereafter referred to as the ‘seed’) using a steel mesh sieve (1.4 mm EFL 2000; Endecotts Ltd., London, England). The dried ground fruit flesh was kept in a sealed and labeled container at 5 °C for further analysis.

### 2.2 Methods for characterisation of fresh blueberry ash fruit

#### 2.2.1 Moisture content

Fruit moisture content was determined by re-weighing the fresh fruit after drying in an oven set at 120 °C for 4 hours. Moisture content was calculated as the weight difference before and after drying and expressed as a mean value and standard deviation.

#### 2.2.2 Fruit size

Twenty fruit were randomly selected, and their diameters were measured using a four decimal thickness gauge. The fruit size (diameter) was expressed as mean value and standard deviation.

#### 2.2.3 Fruit weight

The weight of each fruit from the twenty randomly selected fruit was measured using a four decimal analytical balance. The fruit weight was expressed as mean value and standard deviation.

#### 2.2.4 Flesh weight and seed weight

Ten fruit were randomly selected and the weight of each fruit was measured using a four decimal analytical balance. The fruit flesh was completely removed from the seed, and the weight of each seed was measured. The flesh weight of each fruit was calculated as the weight difference between the whole fruit and its seed. The values are expressed as means and standard deviations.

#### 2.2.5 Seed diameter

The diameter of each seed was then measured using the four decimal thickness gauge.

#### 2.2.6 Fruit colour

The colour characteristics of the blueberry ash fruit were measured using a CR-400 Minolta Chroma Meter (Konica Minolta Ltd., North Ryde, NSW, Australia) calibrated with a white standard tile to determine L* (lightness), a* (redness and greenness) and b* (yellowness and blueness). Chroma (intensity of the colour) and hue angle (H°) (color appearance parameters) were determined from a* and b*, as described previously by Vuong *et al.* [6].

#### 2.2.7 Extractable solid (ES)

The extractable solid was measured by drying 5 mL of the extract at 105 °C for 6 hours. ES is expressed as percentage of extractable solid per gram of dry fruit flesh.

### 2.3 Experimental design for determination of phenolic compounds and antioxidant capacity

To determine the impact of solvents on extraction yield of phenolic compounds, seven extraction solvents were used for comparison in the current study including: water, ethanol, acetonitrile, acetone, ethanol 50%, acetonitrile 50%, and acetone 50%. Ultrasound-assisted extraction was applied to extract the ground dried sample at a solvent-to-sample ratio of 100:1 mL/g of dried sample. The extraction chamber was completely immersed into an ultrasonic bath (Soniclean, 220 V, 50 Hz and 250 W, Soniclean Pty Ltd., Thebarton, Australia) with pre-set conditions for temperature of 35 °C, time of 30 min and power of 150 W. Agitation was conducted for 3 seconds once every five minutes using a Vortex. When the ultrasonic extraction was completed, the extracts were immediately cooled on ice to room temperature (RT), filtered using a 5 mL syringe fitted with a 0.45 μm cellulose syringe filter (Phenomenex Australia Pty. Ltd., Lane Cove, Australia) and diluted to the required volume for quantitative analysis.

The most effective extraction solvent (50% acetone) was then applied to extract the bioactive compounds from the blueberry ash fruit. The extraction process was conducted as described above. The filtered extract was used for the determination of phenolic compounds, antioxidant properties and scanning major bioactive compounds using HPLC.

### 2.4 Methods for determination of chemical properties

The extracts were diluted 10 times with the respective solvents before analysis of total phenolic content, flavonoids and proanthocyanidins.

#### 2.4.1 Total phenolic content (TPC)

TPC was determined as previously described by Vuong *et al.* [10]. 5 mL of 10% (v/v) Folin–Ciocalteu reagent was added to 1 mL of the diluted sample. The mixture was added to 4 mL of 7.5% (w/v) Na_2_CO_3_, mixed well on a vortex vibrator for 2 min and incubated in the dark at room temperature (RT) for 1 h before the absorbance was measured at 760 nm using a UV spectrophotometer (Varian Australia Pty. Ltd., Victoria, Australia). Gallic acid was used as the standard for a calibration curve and the results were expressed as mg of gallic acid equivalents per g of dried sample (mg GAE/g).

#### 2.4.2 Total flavonoids

The total flavonoid content was measured as described by Pham *et al.* [9]. 2 mL of H_2_O was mixed with 0.15 mL of 5% (w/v) NaNO2. The mixture was added to 0.5 mL of the diluted sample and left at RT for 6 min. Then 0.15 mL of 10% (w/v) AlCl_3_ was added and left at RT for 6 min. It was followed by the addition of 2 mL of 4% (w/v) NaOH and 0.7 mL of H_2_O. This final solution was mixed well and left at RT for a further 15 min before the absorbance was measured at 510 nm. Rutin was used as the standard for a calibration curve and the results were expressed as mg of rutin equivalents per gram of dried sample (mg RUE/g).

#### 2.4.3 Proanthocyanidins

The content of proanthocyanidins was determined as previously described by Vuong *et al.* [10]. 3 mL of 4% (w/v) of vanillin was added to 0.5 mL of the diluted sample. The mixture was then added to 1.5 mL of concentrated HCl and left at RT for 15 min before measurement of the absorbance at 500 nm. Catechin was used as the standard for a calibration curve and the results were expressed as mg of catechin equivalents per gram of dried sample (mg CE/g).

#### 2.4.4 Total anthocyanin content

Total anthocyanin content was determined using the spectrophotometric pH differential method as described by Lee *et al.* [11]. The results were expressed as mg of cyanidin-3-glucoside equivalents per g of the dried extract (mg CGE/g).

### 2.5 Methods for determination of antioxidant properties

The extracts were diluted 10 times for DPPH and FRAP assays, and diluted 20 times for CUPRAC assay.

#### 2.5.1 DPPH radical scavenging activity

The radical scavenging activity was measured based on the method described by Thaipong *et al.* [12] with some modifications. A stock solution was prepared by dissolving 12 mg of DPPH with 50 mL of methanol and then storing the solution at −20 °C for further use. The working solution was freshly prepared by mixing the stock solution with methanol until absorbance at 515 nm of 1.1 ± 0.02. 3.8 mL of the working solution was added to 0.2 mL of the sample and then left in darkness at RT for 3 h before measuring the absorbance at 515 nm using the UV spectrophotometer. Trolox was used as the standard for a calibration curve and the results were expressed as mM of trolox equivalents per g of dried sample (mM TE/g).

#### 2.5.2 Cupric reducing antioxidant capacity (CUPRAC)

CUPRAC was determined as previously described by Apak *et al.* [13]. 1 mL of neocuproine, 1 mL of NH4Ac and 1.1 mL of the sample were added to 1 mL of CuCl_2_, mixed and then incubated at RT for 1.5 h before measuring the absorbance at 450 nm. Trolox was used as the standard for a calibration curve and the results were expressed as mM of trolox equivalents per g of dried sample (mM TE/g).

#### 2.5.3 Ferric reducing antioxidant power (FRAP)

FRAP was measured as previously described by Thaipong *et al,.* [12] with some modifications. A working FRAP solution was prepared by mixing 300 mM acetate buffer, 10 mM TPTZ (2, 4, 6-tripyridyl-s-triazine) in 40 mM HCl, and 20 mM FeCl_3_ in the ratio of 10:1:1 and incubating the solution at 37 °C in a water bath (Ratek, Ratek Instruments Pty. Ltd., Victoria, Australia) before use. The sample (0.15 mL) was added to 2.85 mL of the working FRAP solution and incubated at RT in the dark for 30 min before measuring its absorbance at 593 nm. Trolox was used as a standard and the results were expressed as mM trolox equivalents per gram of dried sample (mM TE/g).

### 2.6 HPLC analysis for scanning for major bioactive compounds

A Shimadzu HPLC system (Shimadzu Australia, Rydalmere, NSW, Australia) was used to scan for major bioactive compounds in the blueberry ash fruit extract. An injection volume of 20 μL sample was applied on a 250 mm × 4.6 mm Synergi 4 μ Polar-RP 80A column (Phenomenex Australia Pty. Ltd., Lane Cove, NSW, Australia) with a mobile phase flow rate of 1 mL/min. The mobile phases consisted of two solvent systems, A and B. Solvent A was 0.02% (v/v) orthophosphoric acid, while solvent B was 100% acetonitrile. A gradient elution schedule was used as follows: 100% A from 0 to 5 min; a linear gradient from 100% A to 40% B from 5 to 25 min; then from 40% B to 55% B from 25 to 35 min and remained at 55% B to 45 min; finally from 55%B to 100% A from 45 to 50 min. A UV-Vis detector was used to detect the major bioactive compounds at 380 nm.

### 2.7 Statistical analyses

The one-way ANOVA and the LSD post-hoc test were conducted using SPSS statistical software version 20. Differences between the mean levels of the components in the different experiments were taken to be statistically significant at *p* < 0.05. Correlations between bioactive compounds and antioxidant properties were calculated based on the linear correlation plots of the total polyphenol content (TPC) as a function of each antioxidant capacity.

## 3 Results and discussion

### 3.1 Characteristics of blueberry ash (*Elaeocarpus reticulatus*) fruit

The characteristics of blueberry ash including moisture content, fruit size, fruit weight, flesh (edible portion), seed size and seed weight as well as fruit colour were examined and the results are shown in Table 1. Blueberry ash fruit has a round-oval drupaceous shape. The fruit was dark blue in colour, with a hue angle of 250. The fruit was small in size, with a mean diameter of around 7 mm. Compared with blueberry cultivars Biloxi and Sharpblue, grown as a commercial crop in Guasca (Colombia) (90–150 mm) [14], the fruit of blueberry ash is more than 10 times smaller. It should be noted that blueberry ash belongs to the family Elaeocarpaceae, whereas blueberry cultivars such as *Biloxi* and *Sharpblue* belong to the *Vaccinium* genus, which is in the Ericaceae family [14]. These findings showed that blueberry ash fruit are much smaller than commercially cultivated blueberry fruit.

**Table 1:** Blueberry ash (*Elaeocarpus reticulatus*) fruit characteristics.

Characteristics	Values
Moisture (%)	51.71 ± 2.25
Fruit size (diameter) (mm)	6.81 ± 0.29
Fruit weight (g)	0.21 ± 0.02
Flesh weight (g)	0.14 ± 0.03
Seed weight (g)	0.07 ± 0.01
Seed diameter (mm)	4.99 ± 0.19
Fruit colour	
Lightness	26.16 ± 2.53
Chroma	7.89 ± 1.25
Hue angle	253.67 ± 10.41

The values are the mean ± standard deviation.

Blueberry ash fruit also has a small weight (approximately 0.2 g) and contains a relatively large round seed with a diameter of around 5 mm. The seed weight accounts for 34% of fruit weight. The edible flesh of the fruit is 2 mm thick, however it accounts for approximately 66% of total fruit weight. The moisture content of the flesh was around 50% of the fruit weight. These findings differ slightly to those of Gosper *et al.* [2], who found that the wet weight of blueberry ash flesh was 0.25 g and moisture content was 59%. The difference between the studies can be explained by the influence of other factors such as differences in the collecting location (i.e. soils and climate) and populations (natural versus planted). Compared to commercially cultivated blueberry fruit (80–86%) [15], the moisture content of blueberry ash fruit was much lower. In addition, the weight of blueberry ash was less than three times lighter than commercially cultivated blueberry fruit [15].

### 3.2 Influence of solvents on extractable solids, phenolic compounds and antioxidant properties of blueberry ash

**Fig. 2:**
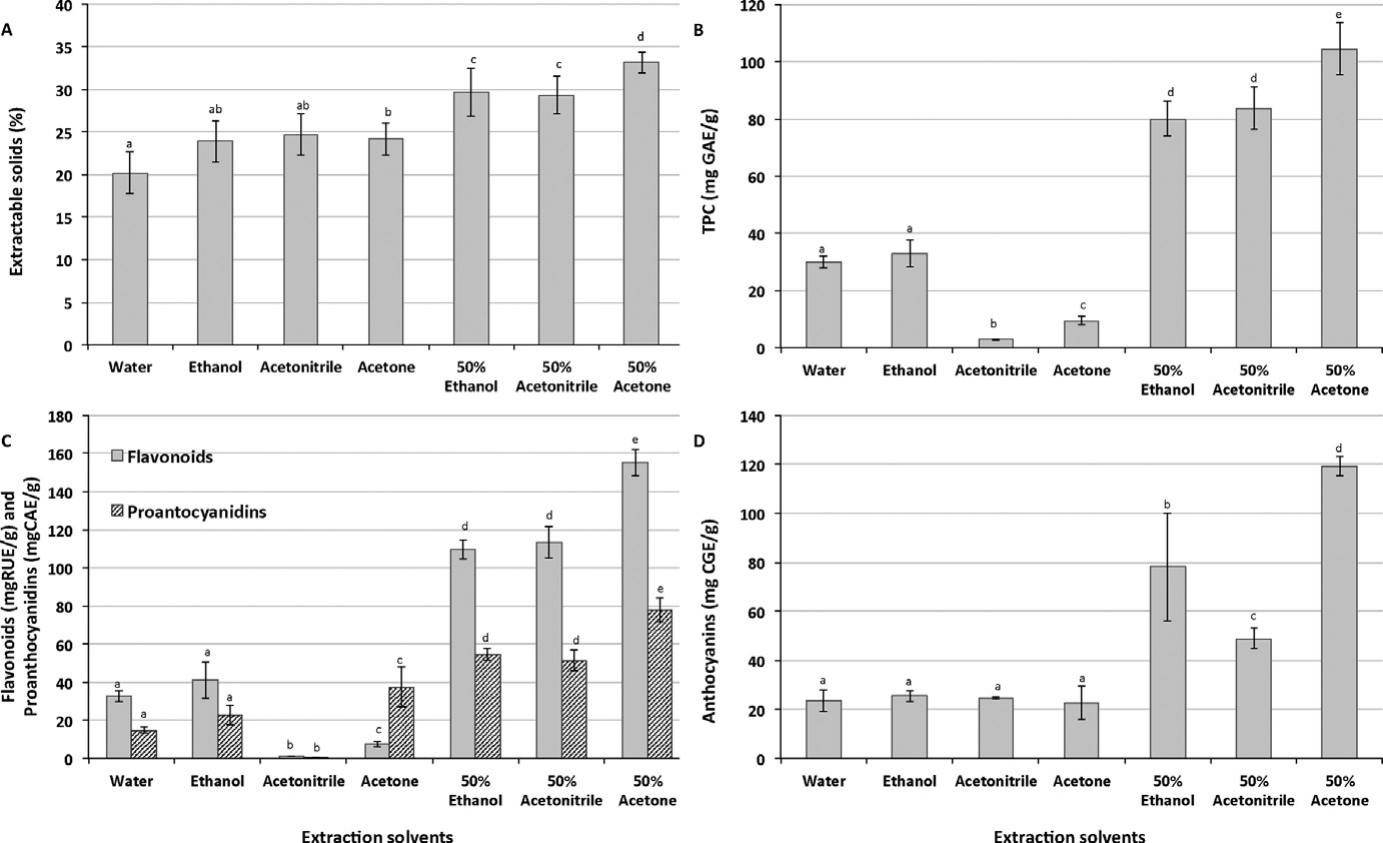
Impact of solvents on extractable solids (A), phenolic content (B), flavonoids and proanthocyanidins (C), and anthocyanins (D) of blueberry ash fruit. The values are the mean of three replications for each solvent ± standard deviation. Columns not sharing the same superscript letter are significantly different at p < 0.05.

It is important to determine the impact of solvents on extractability of extractable solids, phenolic compounds and antioxidant properties of blueberry ash in order to select the most suitable solvent for identification of phenolic compounds and antioxidant properties of this fruit. Results (Fig. 2) showed that levels of extractable solids, total phenolic content, flavonoids, proanthocyanidins and anthocyanins of blueberry ash were significantly affected by extraction solvents.

Extractable solid content (ES) is linked with recovery of soluble compounds for further processing. ES of blueberry ash ranged from approximately 20 to 33% depending on the solvent used (Fig. 2A). Overall, organic solvents gave higher extractable solid content than that of water. However, combining organic solvents with water gave significantly higher content of extractable solids in comparison with water or organic solvent alone (Fig. 2A). Furthermore, 50% acetone produced the highest extractable solid content. These findings were similar to the results of a previous study on *Limnophila aromatica* plants [8]. However, the findings were different to the results of another study on *Helicteres hirsuta* Lour. leaves [9]. Differences between these studies can be explained by the variation in solubility of extractable compounds, such as proteins, pectins, vitamins, minerals and phytochemicals in the samples.

Phenolic compounds are well-known for their link with health benefits. For blueberry ash, total phenolic content (TPC) was significantly influenced by the extraction solvent used (Fig. 2B). Water and ethanol had similar levels of TPC, whereas absolute acetonitrile and acetone had significantly lower levels of TPC, and the combination of water with these organic solvents had significantly higher levels of TPC than water or absolute ethanol alone. Of these, 50% acetone provided the highest level of TPC, which was three times greater than that extracted by water. These findings were in agreement with the results of a previous study on macadamia skin [6]. However, they are different to the results of other studies on *Limnophila aromatica* plants [8] and *Helicteres hirsuta* Lour. leaves [9]. The difference in results can be explained by the variation in the solubility of phenolic compounds in different types of sample.

Flavonoids also varied depending on the types of extraction solvents (Fig. 2C). Water and ethanol could extract more flavonoids from the blueberry ash fruit than absolute acetonitrile or acetone. However, the combination of water and organic solvents including ethanol, acetonitrile or acetone could significantly increase extraction efficiency of flavonoids from blueberry ash. 50% acetone was found to be the best extraction solvent for flavonoids (155 mgRUE/g). These results were similar to that of a previous study on papaya cultivars, which showed that 50% acetone, 50% ethanol and 50% methanol had higher extraction yields of flavonoids than that of absolute acetone, ethanol and methanol as well as water [7].

Proanthocyanidins are classified as condensed tannins and this study found that their extractability was significantly affected by the extraction solvent used (Fig. 2C). Except for acetonitrile, which had the lowest level of proanthocyanidins, absolute organic solvents had significantly higher levels of proanthocyanidins than water. The combination of water and organic solvents also gave the highest extraction efficiency for proanthocyanidins. Of those, 50% acetone gave the highest extraction yield with approximately 155 mg CAE/g. These findings were supported by a previous study on macadamia skin [6].

*Anthocyanins* are water-soluble pigments, which are responsible for most of a fruits colour, and there have been more than 635 anthocyanins identified in nature [16]. Anthocyanins have been linked with various health benefits [17]. The results (Fig. 2D) showed that levels of anthocyanins were significantly affected by the extraction solvent used. Levels of anthocyanins in blueberry ash fruit ranged from 22 to 119 mg CGE/g. Water extraction produced a similar level of anthocyanins to absolute organic solvents; however these levels were significantly lower than those where the solvents were a mixture of water and organic solvents. 50% acetone gave the highest level of anthocyanins and, therefore, this solvent is recommended for extraction of anthocyanins.

**Fig. 3:**
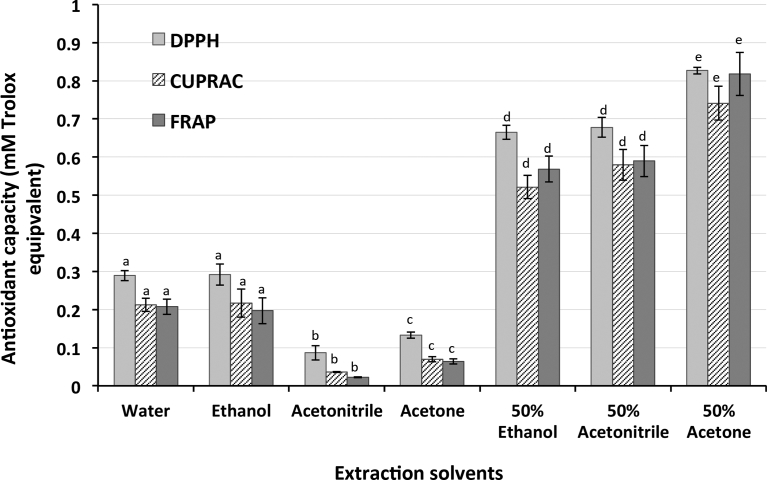
Impact of solvents on antioxidant properties of blueberry ash. The values are the mean of three replications for each solvent ± standard deviation. Columns not sharing the same superscript letter are significantly different at p < 0.05.

The antioxidant capacity of a sample is known as a useful indicator of potential health benefits because it can imply the ability of redox active molecules to scavenge free radicals [18]. However, the antioxidant capacity of a sample can vary depending on the assay used [18]. Therefore, the current study applied three different antioxidant assays including DPPH, CUPRAC and FRAP to determine the impact of extraction solvents on antioxidant capacity of blueberry ash. Fig. 3 showed that the results from the three antioxidant assays were consistent and that different solvents were found to significantly affect the antioxidant capacity of blueberry ash. Absolute acetonitrile and acetone gave the lowest antioxidant capacity, followed by water and ethanol, and then 50% ethanol and 50% acetonitrile. 50% acetone gave the highest antioxidant capacity with approximately 0.8 mM Trolox equivalent.

The results (Table 2) revealed that phenolic compounds and their secondary metabolites -flavonoids and proathocyanidins, as well as anthocyanins, were strongly related to antioxidant capacity, suggesting that these groups of compounds are the major antioxidants in blueberry ash. Further study to isolate these compounds from this sample is recommended.

**Table 2:** Correlation between bioactive compounds and antioxidant properties.

	R square			
	TPC	Flavonoids	Proanthocyanidins	Anthocyanins
DPPH	0.9855	0.9877	0.7549	0.7837
CUPRAC	0.9944	0.9952	0.7697	0.7952
FRAP	0.9899	0.9951	0.7893	0.8315

In general, 50% acetone was found to be the most effective solvent for extraction of phenolic compounds with high antioxidant capacity. According to the guidance for industry provided by the U.S. Food and Drug Administration (FDA) [19], organic solvents used for extraction have been classified into classes 1, 2 or 3. Acetone belongs to class 3, which is regarded as less toxic and of lower risk to human health. Class 3 includes no solvent known as a human health hazard at levels normally accepted in pharmaceuticals. It is considered that amounts of these residual solvents of 50 mg per day or less would be acceptable without justification. Higher amounts may also be acceptable provided they are realistic in relation to manufacturing capability and good manufacturing practice (GMP) [19]. Thus 50% acetone is the solvent of choice as a safe solvent for extraction chemistry and was used for characterisation of blueberry ash fruit. It should be noted that the acetone can be in different forms and grades, such as acetone FCC, acetone reagent, acetone spectrophotometric grade, acetone HPLC grade, and acetone histological grade. As the extract is used for human consumption, acetone FCC is recommended to be use as the FDA has determined it is safe for use as an indirect food additive in adhesives and food-contact coatings and is regarded as a Generally Recognized as Safe (GRAS) substance at certain concentrations.

### 3.3 Determination of phenolic compounds and antioxidant properties of blueberry ash (*Elaeocarpus reticulatus*) fruit

Using the most suitable solvent (50% acetone), the chemical and antioxidant characteristics of blueberry ash fruit were further identified and the results are shown in Table 3. Approximately a third (33%) of the dried fruit weight can be recovered after extraction, revealing that the fruit contained a high level of extractable solids, and a high recovery yield of crude extract could be obtained if using fruit for further processing. Blueberry ash fruit contained approximately 104 mg GAE/g. In comparison with other Australian native fruits, blueberry ash has a lower content of phenolic compounds than Kakadu plum (158 mgGAE/g), but higher than quandong (90 mgGAE/g), Davidson's plum (50 mgGAE/g) and Illawarra plum (68 mgGAE/g) [20, 21].

**Table 3:** Phenolic compounds and antioxidant capacity of Blueberry ash fruit.

Characteristics	Values
Extractable solids (%)	33.18 ± 1.22
Phenolic content (mg GAE/g)	104.47 ± 9.01
Flavonoids (mg RUE/g)	155.21 ± 6.99
Proanthocyanidins (mg CAE/g)	77.96 ± 6.10
Anthocyanins (mg CGE/g)	119.18 ± 4.05
DPPH (mM TE/g)	0.83 ± 0.01
CUPRAC (mM TE/g)	0.74 ± 0.04
FRAP (mM TE/g)	0.82 ± 0.06

The values are the mean of at least triplicate ± standard deviation.

**Fig. 4:**
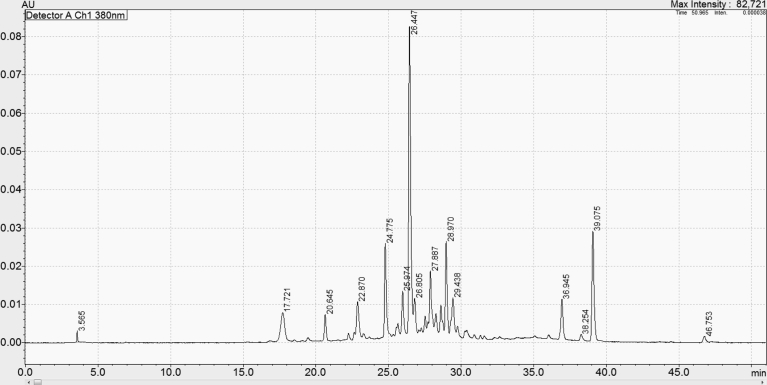
HPLC chromatogram of blueberry ash fruit extract detected using a VUV-Vis detector at 380 nm. Each peak presents a bioactive compound in blueberry ash.

Blueberry ash fruit had levels of flavonoids and proanthocyanidins of 155 mg RUE/g and 77 mg CAE/g, respectively. These levels were higher than those of agen prune (59 and 3.22 mg/100 g, respectively), apricots (56 and 2 mg/100 g, respectively), and figs (104 and 2 mg/100 g, respectively) [22]. In addition, blueberry ash fruit had high levels of anthocyanins (119 mg CGE/g), which are responsible for the dark blue of the fruit. Blueberry ash fruit had higher anthocyanins than those of blueberry (38 mg/g), lowbush (wild) blueberries (*Vaccinium angustifolium*) (10 mg/g) and highbush blueberries (Rubel variety) (*Vaccinium corymbosum*) (15 mg/g) and riberry (35 mg/g) [21, 23]. Results from HPLC scanning at 380 nm showed that there were over 12 major individual bioactive compounds could be detected from the extract of blueberry ash fruit at 380 nm (Fig. 4). As previously reported, anthocyanins in blueberries could be well detected at 510 nm and 380 nm [24], suggesting that these major bioactive compounds could be responsible for major anthocyanins, the pigment compounds of the fruit. Further study is recommended to identify these compounds. Blueberry ash fruit contained high antioxidant capacity. The antioxidant capacity of blueberry ash was higher than that found in blueberry and riberry, as well as in other Australian native fruits such as quandong and Davidson's plum [21]. It should be noted that vitamin C, a common vitamin found in many fruits, could be contributing to the antioxidant activity of blueberry ash. Future research needs to analyse the level of vitamin C, as well as its contribution to total antioxidant capacity of blueberry ash. Nevertheless, there is great potential for blueberry ash fruit to be further tested for its link with health benefits.

## 4 Conclusions

This study found that blueberry ash fruit has a high portion of flesh, which could be of potential use as human food as well as for further processing. This fruit (flesh) contained high levels of total phenolic content, flavonoids, and proanthocyanidins. This fruit also contains a high level of anthocyanins, which contribute to the dark blue colour of the fruit. All of these compounds contribute to the antioxidant capacity of this fruit, which was higher than a number of other fruits, including Australian native fruits, and they have potential for health benefits. This study also found more than 12 major individual compounds in this fruit and they could be responsible for antioxidant and biological properties of this fruit. Therefore, further studies are recommended to isolate and identify these individual compounds and test their potential use in the food or pharmaceutical industries.

## Declarations

### Author contribution statement

Quan V Vuong, Hong Ngoc Thuy Pham, Hang T. Vu, Trung Thanh Dang, Thanh Van Ngo: Conceived and designed the experiments; Performed the experiments; Analyzed and interpreted the data; Wrote the paper.

Anita C. Chalmers: Performed the experiments; Analyzed and interpreted the data.

### Funding statement

This research did not receive any specific grant from funding agencies in the public, commercial, or not-for-profit sectors.

### Competing interest statement

The authors declare no conflict of interest.

### Additional information

No additional information is available for this paper.
